# MarR-Dependent Transcriptional Regulation of *mmpSL5* Induces Ethionamide Resistance in Mycobacterium abscessus

**DOI:** 10.1128/aac.01350-22

**Published:** 2023-03-29

**Authors:** Ronald Rodriguez, Nick Campbell-Kruger, Jesus Gonzalez Camba, John Berude, Rachel Fetterman, Sarah Stanley

**Affiliations:** a Department of Plant & Microbial Biology, University of California, Berkeley, California, USA; b Department of Molecular & Cell Biology, University of California, Berkeley, California, USA

**Keywords:** *Mycobacterium abscessus*, nontuberculosis mycobacteria (NTM), antibiotic resistance, ethionamide, transcriptional regulation

## Abstract

Mycobacterium abscessus (*Mabs*) is an emerging nontuberculosis mycobacterial (NTM) pathogen responsible for a wide variety of respiratory and cutaneous infections that are difficult to treat with standard antibacterial therapy. *Mabs* has a high degree of both innate and acquired antibiotic resistance to most clinically relevant drugs, including standard anti-mycobacterial agents. Ethionamide (ETH), an inhibitor of mycolic acid biosynthesis, is currently utilized as a second-line agent for treating multidrug-resistant tuberculosis infections. Here, we show that ETH displays activity against clinical strains of *Mabs* *in vitro* at concentrations that are >100× lower than other mycolic acid targeting drugs. Using transposon mutagenesis followed by transposon sequencing (Tn-Seq) and whole-genome sequencing of spontaneous ETH-resistant mutants, we identified *MAB_2648c* as a genetic determinant of ETH sensitivity in *Mabs. MAB_2648c* encodes a MarR family transcriptional regulator of the TetR class of regulators. We show that *MAB_2648c* represses expression of *MAB_2649* (*mmpS5*) and *MAB_2650* (*mmpL5*). Further, we show that derepression of these genes in *MAB_2648c* mutants confers resistance to ETH, but not other antibiotics. To identify determinants of resistance that may be shared across antibiotics with distinct mechanisms of action, we also performed Tn-Seq during treatment with amikacin and clarithromycin, drugs currently used clinically to treat *Mabs.* We found very little overlap in genes that modulate the sensitivity of *Mabs* to all three antibiotics, suggesting a high degree of specificity for resistance mechanisms in this emerging pathogen.

## INTRODUCTION

Mycobacterium abscessus (*Mabs*) is an emerging, nontuberculosis mycobacterial (NTM) pathogen that has become prevalent in individuals suffering from various underlying lung disorders, including bronchiectasis, chronic obstructive pulmonary disorder (COPD), and cystic fibrosis (1, 2). In the United States, *Mabs* infections represent up to 13% of all NTM pulmonary infections, second only to infections with the Mycobacterium avium complex (MAC) (3, 4). The epidemiology of *Mabs* infections has expanded globally in the past decade, representing up to 35% of clinical NTM cases in some regions (5, 6). These infections are difficult to treat with standard antibacterial therapy, with cure rates ranging from 30 to 50%, making *Mabs* one of the most antibiotic resistant pathogens in clinical settings (7). A better understanding of the biological mechanisms that allow *Mabs* to resist antibiotic action, in combination with drug development and repurposing efforts, is necessary for improving the therapeutic success of *Mabs* infections (8, 9).

Current treatment of *Mabs* infections in health care settings requires a minimum of 12 months of intensive antibacterial therapy, which often fails (10). In pulmonary cases, lung resection or transplantation is often necessary (11). Antibiotic treatment options include intravenous or inhaled amikacin (aminoglycoside) in combination with clarithromycin or azithromycin (macrolides), imipenem (β-lactam), cefoxitin (β-lactam), tigecycline (tetracycline), moxifloxacin (fluoroquinolone), and linezolid (oxazolidinone) (10, 12). Resistance to currently administered antibiotics is common and a significant contributor to treatment failure (13). A unique feature of the *Mabs* resistance profile is that the bacteria are intrinsically resistant to most clinically relevant antibiotics (13). However, only a few intrinsic resistance mechanisms have been identified to date. Antibiotic-modifying monooxygenases and beta-lactamases that contribute resistance to tetracycline and various beta-lactam antibiotics have been identified (14, 15). In addition, the transcription factor Whib7 was found to contribute to the expression of genes that confer resistance to numerous ribosome targeting antibiotics (16). While past studies have been able to identify a few genes that are important for intrinsic resistance, we still lack a complete understanding regarding the remarkable breadth of resistance observed in *Mabs*. The *Mabs* genome contains numerous putative phosphotransferases, acetyltransferases, and transporters that could modify and efflux antibiotics (17, 18), but experimental evidence for how these mechanisms relate to specific antibiotics is lacking. It is also unclear whether there are a few universal mechanisms that regulate resistance to antibiotics broadly, or whether there are numerous drug-specific resistance mechanisms.

Mycolic acids are known to play critical roles in the pathogenesis, virulence, and impermeability of mycobacteria, and targeting the mycolic acid biosynthetic pathway has yielded several successful anti-mycobacterial agents (19–21). The drug isoniazid (INH) targets mycolic acid biosynthesis by inhibiting the NADH-dependent enoyl ACP reductase InhA, a component of FAS-II (19, 22). INH is the cornerstone of antituberculosis therapy, but has little efficacy against *Mabs* (23–25). The reasons for the lack of efficacy of INH against *Mabs* are unclear (25). Thiacetazone (THZ), an inhibitor of the HadABC dehydratase complex associated with FAS-II, is only modestly active against *Mabs in vitro*, but chemical derivatives of THZ have shown improved efficacy (26, 27). These findings suggest that mycolic acid inhibition may represent an untapped research area for developing new drug regimens against *Mabs* infections.

Ethionamide (ETH), a structural analog of INH, is a second-line mycolic acid inhibitor currently used to treat drug-resistant infections associated with Mycobacterium tuberculosis (*Mtb*) (28). Similar to INH, ETH inhibits mycolic acid biosynthesis in *Mtb* by inhibiting InhA (23). ETH requires activation by EthA, a flavin-containing monooxygenase, that covalently links NAD to ETH (ETH-NAD) (29, 30). ETH-NAD acts as a competitive inhibitor by competing with NADH for binding in the active site of InhA (30). ETH resistance is common among *Mtb* clinical isolates (31). Mutations in *ethA* reduce ETH bioactivation, conferring resistance (23). Here, we show that clinical isolates of *Mabs* are susceptible to ETH in axenic culture at low micromolar concentrations. Further, we show that combining ETH with other clinically used antibiotics results in suppression of resistance emergence.

Tn-Seq is a powerful tool that facilitates the identification of genes important for bacterial growth under any condition in a high-throughput manner (32). Tn-Seq has been previously used to identify genes important for growth during antibiotic exposure in Pseudomonas aeruginosa, Staphylococcus aureus, and Mycobacterium avium (33–35). Tn-Seq libraries have been previously generated in *Mabs* and used to explore essential genes for growth in axenic culture, as well as genes required for survival in a lung epithelial model (36–38). Here, we use Tn-Seq to identify transposon mutants that display both growth defects and growth advantages when exposed to the ETH. We identify *MAB_2648c*, a *marR* homolog in *Mabs*, as an important genetic determinant of ETH sensitivity. Point mutations and deletion of *MAB_2648c* confer ETH resistance. This increase in resistance is due to upregulation of *mmpL5 and mmpS5*, which encode two putative transport proteins that are part of larger families of proteins (MmpS and MmpL proteins) known to confer resistance to other antibiotics in *Mabs* (27, 39). Deletion of these genes re-sensitizes ETH-resistant bacteria. Interestingly, upregulation of *mmpS5* and *mmpL5* does not appear to confer broad antimicrobial resistance. Indeed, comparison of Tn-Seq results across the antibiotics ETH, amikacin (AMK), and clarithromycin (CLR) reveals a surprising lack of overlap, suggesting a high degree of specificity in mechanisms that modulate antibiotic susceptibility/resistance in this important pathogen.

## RESULTS

### Construction and characterization of a transposon library in *Mabs*.

**FIG 1:**
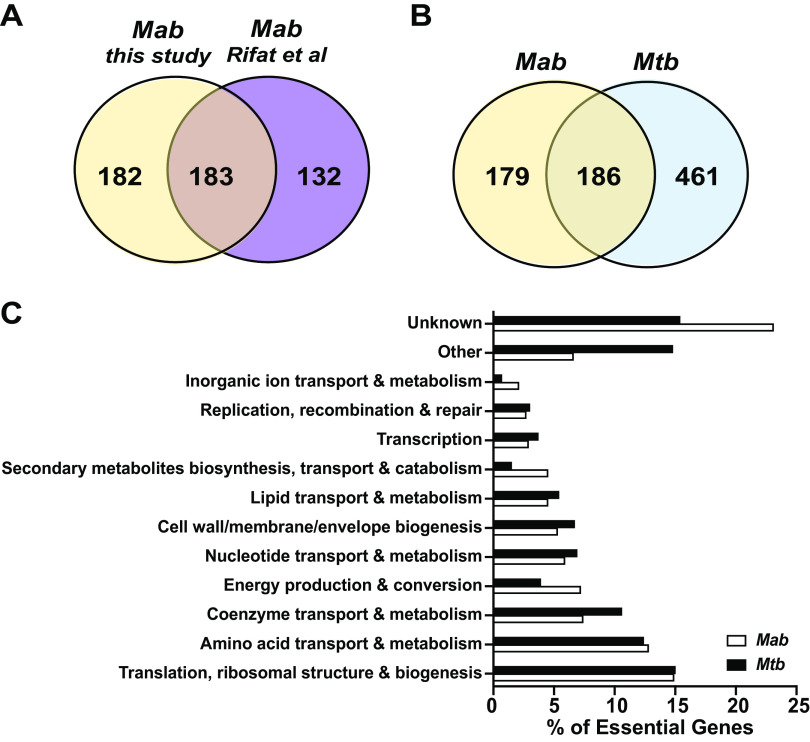
Tn-Seq analyses of the essential genome in *Mabs*. (A) Venn diagram displaying essential genes identified in this study compared with Rifat et al. (B) Venn diagram displaying essential gene homologs in *Mabs* and *Mtb*. (C) Essential genes in *Mabs* were grouped in cluster of orthologous groups (COG) and then compared to the essential genome in *Mtb*. “Other” represents COG categories containing less than 2% of the essential genome. “Unknown” represents genes without any COG annotations.

To enable genetic screening in *Mabs*, we constructed a transposon mutant library in WT *Mabs* ATCC 19977 by packaging the *Himar1* transposon in phage ΦMycoMarT7 followed by bacterial transduction (40). We chose this strain since it has a fully sequenced genome and is genetically tractable (41, 42). To determine the location and abundance of transposon insertions in the resulting library, we collected genomic DNA and sequenced the transposon junctions (43, 44). We obtained approximately 80,000 unique transposon insertions in nonessential genes. We were not able to obtain insertions in 376 genes, suggesting that these genes are essential (Table S1). Of these genes, 183 overlapped with a previous study that identified essential genes in *Mabs* using TnSeq (37). Using only genes identified as essential in our study, 186 genes in *Mabs* overlaps with the essential genome of *Mtb* (Fig. 1B). In *Mabs*, genes associated with translation, ribosomal structure and biogenesis was the most represented cluster of known orthologous group (COG) category among those without insertions (Fig. 1C). Overall, the trends in COG categories were similar when comparing *Mtb* and *Mabs*. As expected, genes encoding components of mycolic acid biosynthesis were predicted to be essential (Table S1).

### Mycolic acid inhibitors are largely ineffective against *Mabs*, although ETH activity displays promising repurposing potential.

**FIG 2:**
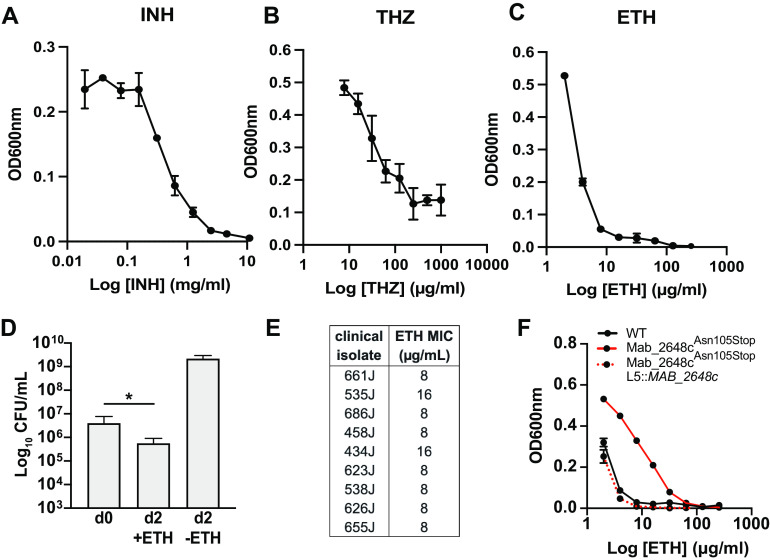
ETH displays mild bactericidal activity against *Mabs*. Dose-response curves for (A) INH, (B) THZ, and (C) ETH against WT *Mabs*. (D) WT *Mabs* was treated with and without ETH (20 μg/mL) for 2 days and viable bacteria were enumerated by CFU (E) ETH MIC values for clinical isolates of *Mabs*. (F) ETH dose-response curves for WT, MAB_2648c ^Asn105Stop^, and MAB_2648c ^Asn105Stop^ complement (MAB_2648c ^Asn105Stop^ L5:: *MAB_2648c*) strains. All experiments are representative of at least three biological replicates except for (E), which is representative of two biological replicates. Error bars represent standard deviation, *P ≤ *0.05 (*).

To test antimicrobial activity of mycolic acid inhibitors against *Mabs*, we determined the MIC of representative mycolic acid inhibitors: isoniazid (INH), thiacetazone (THZ), and ETH (23, 26, 27), against WT *Mabs*. We chose INH and ETH because they possess clinical relevance in the context of *Mtb* and their mechanism of action has been extensively studied (23, 31, 45). The MIC_99_ for INH (1.25 mg/mL) and THZ (>500 μg/mL) are much higher than those commonly observed with drug sensitive *Mtb* and are above standard clinical breakpoints (Fig. 2A and B, Table S3) (46, 47). We did not observe complete growth inhibition with the THZ concentrations tested against *Mabs*, which is consistent with previous work that has reported THZ is largely ineffective against *Mabs* (27). The high-level of intrinsic resistance displayed by *Mabs* to these agents is a major reason why they are not used in clinical settings where *Mabs* infections are observed. Interestingly, the ETH MIC_99_ (8 μg/mL) suggests that this drug is at least 100× more potent than THZ or INH against *Mabs in vitro* (48). Indeed, we found that ETH at this concentration displays mildly bactericidal activity against *Mabs* (Fig. 2D). Importantly, the ETH MIC of 9 clinical isolates tested were close to our WT ATCC 19977 strain, suggesting that relative sensitivity to ETH may not be a unique feature of a single strain (Fig. 2E). This is in contrast to what has been observed with other clinical isolates of *Mabs*, in which the ETH MIC ranged from 16 to 256 μg/mL (49). Therefore, the degree of ETH activity observed here against *Mabs* may be not representative of all clinical isolates that may also display acquired resistance to ETH.

### Whole-genome sequencing of ETH-resistant bacteria identifies mutations in *MAB_2648c*.

In *Mtb*, ETH resistance in drug-resistant clinical strains is often conferred by mutations in *ethA* or *inhA* (23). To identify mutations that confer resistance to ETH in *Mabs*, we generated spontaneous resistant mutants by plating wild-type (WT) bacteria on solid media containing 2 and 3× the agar MIC of ETH for *Mabs*, concentrations just below the maximum solubility of ETH in agar medium (Fig. S1A). Three resistant isolates (R128, R150, and R200) were selected for further analysis. First, to validate that the isolated mutants were indeed ETH resistant, we exposed WT and mutant bacteria to a 2-fold dilution series of ETH in broth. We found that the mutants were approximately 8-fold more resistant (MICs: 8 μg/mL in WT and 64 μg/mL in R128, R150, and R200) to ETH than WT. To identify the genetic basis for resistance, we performed whole-genome sequencing of R128, R150, and R200. Only one mutation was identified that was present in mutant bacteria but not in WT, consisting of a GC dinucleotide deletion in *MAB_2648c* at nucleotide positions 354 and 355. This deletion alters the reading frame of *MAB_2648c*, introducing a premature stop codon at amino acid position 105 (*Mab_2648c* ^Asn105Stop^), which was confirmed with Sanger sequencing of PCR-amplified *MAB_2648c*. *MAB_2648c* encodes a putative *marR* (**m**ultidrug **a**ssociated **r**esistance **r**egulator) transcriptional regulator that represents a subtype of TetR transcriptional repressors, many of which have been associated with resistance to functionally diverse antibiotics in different bacteria (50). Introducing an integrative copy of WT *MAB_2648c* in the spontaneous mutants restored ETH sensitivity (Fig. 2F). Many clinical isolates of *Mtb* that display acquired resistance to ETH are cross-resistant to INH due to mutations in the promoter of *inhA* (51). Our isolated *MAB_2648c* mutants do not have increased resistance to INH relative to the parental WT strain (Fig. S1B to D).

### Transposon insertions in *MAB_2648c* confer a growth advantage in the presence of ETH.

**FIG 3:**
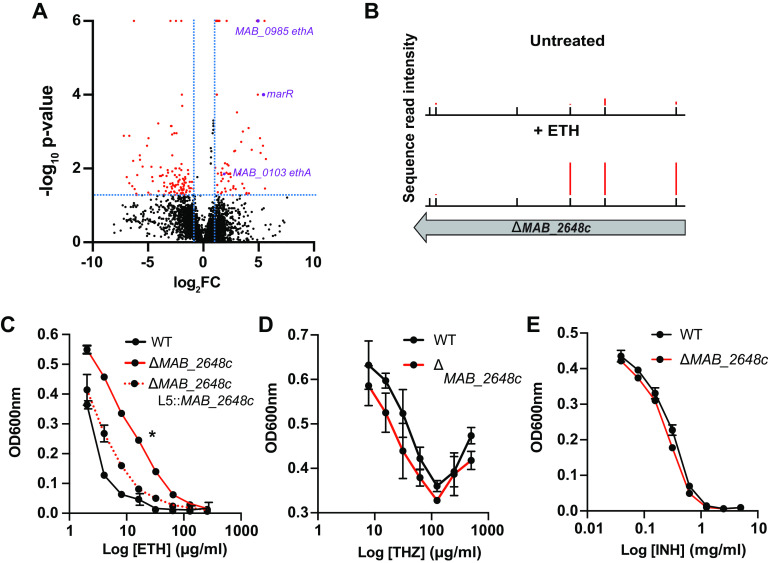
*MAB_2648c* is a determinant of ETH susceptibility. (A) Volcano plot displaying all transposon insertion mutants from a Tn-Seq screen in the presence of ETH. Red dots represent mutants with log fold change >2 and *P* ≤ 0.05 (B) Transposon sequence read densities for *MAB_2648c* in the presence and absence of ETH. Black tick marks represent all possible insertion sites. (C-E) Dose-response curves for WT, Δ*MAB_2648c*, and Δ*MAB_2648c* complement (Δ*MAB_2648c* L5::*MAB_2648c*) strains in the presence of ETH (C), THZ (D), and INH (E). All experiments are representative of at least three biological replicates. Error bars represent standard deviation. *P ≤ *0.05 (*) for Δ*MAB_2648c* versus complemented strain.

To more systematically identify additional genes that modify the efficacy of ETH against *Mabs*, we screened mutants in our transposon (Tn) library for changes in growth in the presence of subinhibitory ETH (Fig. 3A, Fig. S2A). The Tn library was cultured in the presence of ETH for 24 h, at which time surviving bacteria were plated on agar plates. DNA was prepared from resulting colonies and transposon gene junctions were amplified and sequenced as previously described (43, 44); changes in transposon abundance after exposure to ETH were analyzed using TRANSIT (Table S2) (44). We identified 208 genes as potential modulators of susceptibility to ETH using a 2-fold change cutoff and a *P-*value of ≤ 0.05 (Fig. 3A). Among genes with annotations, those associated with transcription were the most abundant, representing approximately 9% of the identified hits (Fig. S2B). Interestingly, mutations in two of the three *ethA* homologs in *Mabs* seemed to confer resistance in the screen (Fig. 3A), suggesting that multiple monooxygenases may contribute to ETH activation in *Mabs*.

In agreement with our data from the generation of spontaneous resistant mutants, we found that bacteria carrying insertions in *MAB_2648c* were approximately 5-fold more abundant in the presence of ETH (Fig. 3A and B). To validate these findings, we constructed a deletion mutant of *MAB_2648c* (Δ*MAB_2648c*) and examined growth in the presence and absence of ETH. We found that Δ*MAB_2648c* was approximately 8-fold more resistant to ETH compared to WT bacteria (MICs: 8 μg/mL for WT and 64 μg/mL for Δ*MAB_2648c*) (Fig. 3C). Thus, using two independent methods, we show that loss of *MAB_2648c* function leads to ETH resistance. Deletion of *MAB_2648c* did not confer resistance to any other antibiotic tested, including INH and THZ (Fig. 3D and E) (Table S3). This result is surprising, as in other bacteria, including E. coli (single MarR regulator) and P. aeruginosa (13 MarR regulators), loss of MarR activity confers resistance to a diversity of antibiotics (52–54).

### *Mab_2648c* negatively regulates expression of *mmpS5-mmpL5* and contributes to ETH resistance.

**FIG 4:**
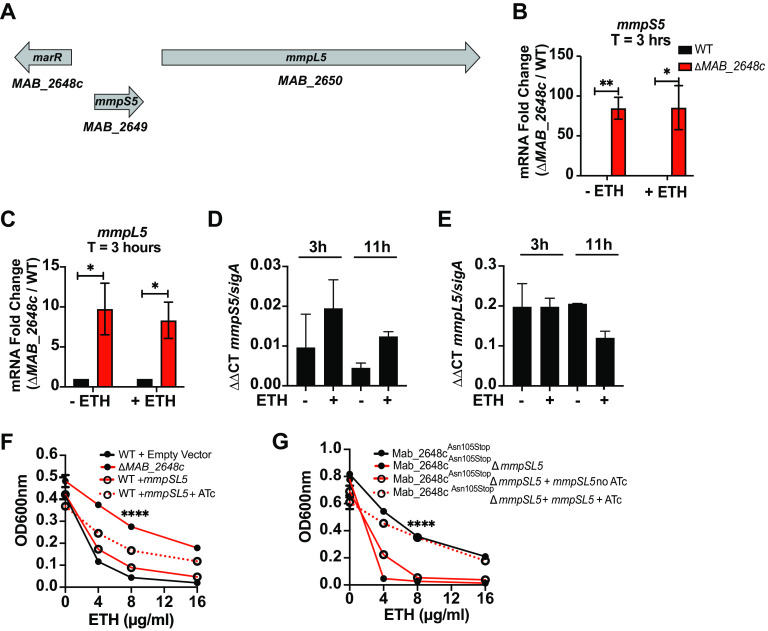
*mmpS5* and *mmpL5* are negatively regulated by Mab_2648c and contribute to ETH resistance. (A) Schematic representation of the genetic organization of *MAB_2648c*, *mmpS5*, and *mmpL5*. (B-E) Gene expression levels of *mmpS5* and *mmpL5* in WT and Δ*MAB_2648c* bacteria in the presence and absence of ETH at the indicated time points. (F-G) ETH dose-response curves for the indicated strains. ATc (100 ng/mL) was included where appropriate. All experiments were performed at least in biological duplicates. Error bars represent standard deviation. *P ≤ *0.05 (*); *P ≤ *0.01 (**); *P ≤ *0.0001 (****) comparing *mmpSL5* mutant with and without ATc.

TetR regulators often negatively regulate expression of target genes that are transcribed divergently from the regulator, separated by an intergenic region of approximately <200 bp (bp) (50). The genes *MAB_2649* (*mmpS5*) and *MAB_2650* (*mmpL5*) are in a putative operon located 369 bp upstream of *MAB_2648c* (Fig. 4A). Mutations in TetR regulators often lead to upregulation of *mmpS* and *mmpL* genes, which contribute to clofazimine, bedaquilline, and THZ resistance in *Mabs* (27, 39). We hypothesized that *mmpS5* and *mmpL5* contribute to ETH resistance, and that in WT bacteria, their expression is repressed by Mab_2648c. To address this hypothesis, we first measured expression levels of *mmpS5* and *mmpL5* by qRT-PCR in WT and Δ*MAB_2648c* bacteria. Regardless of the presence of ETH, expression levels of *mmpS5* were upregulated >80-fold in Δ*MAB_2648c* compared to WT (Fig. 4B). A similar trend was observed for *mmpL5* (Fig. 4C). The addition of ETH to bacterial cultures alone did not affect expression of *mmpS5* (Fig. 4D) or *mmpL5* (Fig. 4E) in WT bacteria, suggesting that Mab_2648c activity is not directly regulated by ETH. To determine whether upregulated expression of *mmpS5* and *mmpL5* in Δ*MAB_2648c* contribute to ETH resistance, we overexpressed both genes in WT bacteria under an anhydrotetracycline (ATc) inducible promoter (55) and examined bacterial growth in the presence of ETH. Overexpression of *mmpS5* and *mmpL5* increased the ETH MIC (>16 μg/mL) compared to bacteria not induced with ATc (Fig. 4F). These data suggest that upregulation of *mmpSL5* is responsible for the ETH resistance phenotype observed in the absence of *MAB_2648c*. To further corroborate this finding, we investigated the contribution of *mmpS5* and *mmpL5* to ETH resistance in Mab_2648c^Asn105Stop^ bacteria. In agreement with our Δ*MAB_2648c* qPCR data, we also found that *mmpSL5* expression is upregulated in Mab_2648c^Asn105Stop^ bacteria compared to WT bacteria (Fig. S3 C-F). Deletion of *mmpSL5* (Δ*mmpSL5)* in this background sensitized bacteria to ETH at least 4-fold compared to Mab_2648c^Asn105Stop^ bacteria (MICs: 4 μg/mL in Δ*mmpSL5* and 16 μg/mL in Mab_2648c^Asn105Stop^ bacteria) (Fig. 4G). These results suggest that the loss of Mab_2648c activity leads to upregulation of *mmpSL5*, whose activity ultimately provides ETH resistance.

We hypothesized that MmpSL5 may function as a transporter that exports ETH, because many membrane transporters function as efflux pumps to export antibiotics (56). To test this hypothesis, we tested whether the Δ*MAB_2648c* mutant strain that expresses *mmpSL5* at high levels displays higher rates of ethidium bromide (EtBr) efflux, an assay commonly used to assay mycobacterial efflux in the context of drug resistance (57). Although we observed EtBr accumulating in *Mabs* cells in a dose dependent manner (Fig. S4), we did not observe any difference in EtBr levels when comparing WT and the Δ*MAB_2648c* mutant at a single dose. The inability to detect increased EtBr efflux in the Δ*MAB_2648c* mutant does not rule out the hypothesis that *mmpSL5* is an ETH transporter, but it suggests there may be specificity to the transport that precludes the use of this assay.

### ETH treatment with CLR or AMK suppresses the emergence of resistant mutants.

**FIG 5:**
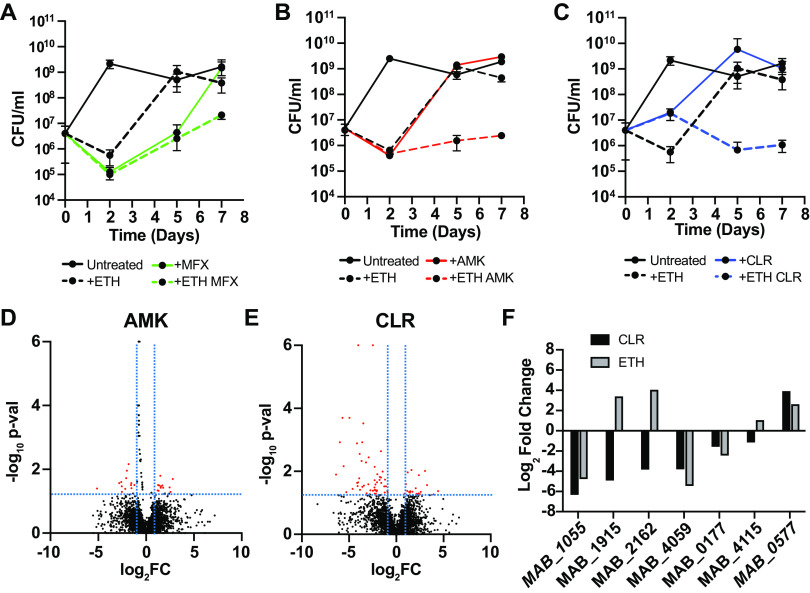
Prolonged ETH treatment potentially suppresses emergence of drug resistance to some clinically relevant antibiotics. (A–C) WT *Mabs* was treated with and without ETH (20 μg/mL) in the presence of moxifloxacin (MFX, 3 μg/mL), amikacin (AMK, 25 μg/mL), and clarithromycin (CLR, 3 μg/mL) or in combination with ETH. At the indicated time points, bacteria were collected, washed, and then 10-fold serial dilutions were plated on LB agar for CFU/mL enumeration. (D–E) Volcano plots displaying all gene hits from a Tn-Seq screen in the presence of AMK (D) and CLR (E). Red dots represent mutants that display growth advantages while black dots represent mutants that display growth defects in the presence of the indicated drug. (F) Fold changes in the transposon read intensities of select mutants from our Tn-Seq screen with ETH and CLR. All experiments were performed in biological duplicates. Error bars represent standard deviation.

Because *Mabs* infections are almost never treated with sole antibiotic regimens (10), we next tested whether ETH synergizes with commonly prescribed antibacterial agents. We tested ETH in combination with three front line drugs for treating *Mabs* infections that are representative of antibiotics with different mechanisms of action: amikacin (AMK, aminoglycoside), clarithromycin (CLR, macrolide), and moxifloxacin (MFX, fluoroquinolone). We chose to test ETH in combination with these antibiotics at concentrations representative of clinically achievable levels (58–60). Treatment with ETH or MFX alone resulted in mildly bactericidal activity during the first 2 days of treatment (Fig. 5A). However, after day 2 bacteria grow in the presence of either antibiotic (Fig. 5A), likely due to the emergence of resistant mutants. Although we did not observe either synergy or antagonism at early time points of treatment with ETH and MFX after 2 days of treatment, there was extensive bacterial growth after day 2, even in the presence of both antibiotics (Fig. 5A). Similar to MFX, treatment with AMK or CLR alone resulted in the rapid emergence of resistant mutants. However, the combination of ETH with either AMK or CLR prevented the outgrowth of bacteria that occurred after 2 days of treatment with single agents (Fig. 5B and C). These data suggest that while synergy was not observed, ETH may have the potential to delay resistance emergence to antibiotics used against *Mabs*.

Δ*MAB_2648c* did not exhibit cross-resistance to other antibiotics tested (Table S3), suggesting a unique function in mediating ETH resistance. This was surprising, as MarR homologs often confer resistance to multiple antibiotics in other bacterial species (52–54). The fact that resistant mutants fail to emerge in the combinatorial treatment of ETH with AMK and CLR suggests that there may be unique mechanisms of resistance to each of these antibiotics. To determine whether there are mediators that confer susceptibility or resistance across multiple antibiotics in *Mabs*, we performed TnSeq in the presence of both AMK and CLR as described for ETH (Table S2). Using the same fold change and significance cutoffs as in our ETH Tn-Seq, we found 28 significant genes in AMK and 70 genes in CLR (Fig. 5D and E, Fig. S5, S6) that may modulate susceptibility to these antibiotics. No genes were similarly significant in both the ETH and AMK conditions, and only 7 genes were important in both the ETH and CLR conditions. Similar gene hits identified in the presence of ETH and CLR are *MAB_1055* (Conserved Hypothetical Peptidase), *MAB_1915* (Probable Fatty Acid CoA Ligase FadD), *MAB_2162* (Putative AAA-family ATPase Mpa), *MAB_4059* (Hypothetical Protein), *MAB_0177* (Antigen 85-A/B/C Precursor), *MAB_4115* (Putative MmpL Membrane Protein), and *MAB_0577* (Putative ABC Transporter Solute Binding Protein). Insertions in three of these genes conferred a growth defect in both conditions, while insertions in only one gene conferred a growth advantage in both conditions (Fig. 5E). Insertions in the remaining three genes had differential effects in the two conditions.

## DISCUSSION

*Mabs* is remarkable among drug-resistant bacterial pathogens in that resistance is intrinsic but can also be acquired to functionally diverse antibiotics (17). Unlike most antibiotics that target mycolic acid biosynthesis that are completely ineffective against *Mabs*, we show here that ETH is mildly bactericidal against *Mabs in vitro*. Using a combination of bacterial genetics, whole-genome sequencing, and transposon mutagenesis, we identified *MAB_2648c* as an important determinant of ETH sensitivity in *Mabs*, as loss of *MAB_2648c* leads to ETH resistance. This loss is associated with upregulation of *mmpSL5*, which contributes to ETH resistance in a *MAB_2648c*-dependent manner. Our findings expand on the current knowledge regarding the role of *mmpSL* genes in conferring antibiotic resistance in *Mabs*. In line with what we have reported here, the loss of other TetR regulators in *Mabs* leads to upregulation of other *mmpSL* genes that confer resistance to bedaquiline, clofazimine, and THZ (27, 39). It is speculated that many of the *mmpSL* genes in *Mabs* encode transporters that efflux these antibiotics into the extracellular space (18), although this requires further evaluation. Whether ETH is being transported by MmpSL5 remains unknown. Interestingly, the TetR regulator Mab_2299c negatively regulates two genetically distant *mmpSL* couples (*MAB_2300-2301* and *MAB_1135c-1134c*) that confer cross-resistance to bedaquiline and clofazimine (39). We did not find any differences in bedaquiline and clofazimine susceptibility in the absence of *MAB_2648c* (Table S3). TetR regulators represents the largest class of transcriptional regulators in *Mabs*, a feature that is characteristic of saprophytic mycobacteria (50). Of the 139 TetR regulators found in the *Mabs* genome, 21 of these are annotated as MarR (61). It is possible that the other MarR regulators confer resistance to antibiotics with diverse modes of action, although this remains to be explored.

We now have several examples of TetR regulators conferring drug resistance in *Mabs*, highlighting the importance of deciphering the roles of those that have yet to be studied in the context of drug resistance (27, 39). Many of these findings have been obtained through the generation of laboratory, drug-resistant strains and have yet to be extended to clinical isolates (27, 39). Sequencing of TetR regulators in drug-resistant clinical isolates could help us identify novel genetic determinants of drug resistance. In our ETH Tn-Seq screen, we identified Tn mutants with insertions in 17 TetR regulators (Table S2). Tn mutants with insertions in three other genes besides *MAB_2648c* (*MAB_2885*, *MAB_2731*, and *MAB_0979*) displayed a growth advantage in the presence of ETH while mutants with insertions in the other 13 TetRs displayed a growth defect (Table S2). In our Tn-Seq screen with AMK, we identified Tn insertions in 2 TetR regulators: *MAB_2061c* (growth defect) and *MAB_4026c* (growth advantage) (Table S2). Mutants with Tn insertions in two additional TetR regulators (*MAB_1881c* and *MAB_2952c*) displayed growth defects in our Tn-Seq screen with CLR (Table S2). These Tn-Seq hits can be utilized to expand our knowledge on the contribution of TetR regulators as being important genetic determinants for growth on clinically relevant antibiotics.

Questions regarding the role of Mab_2648c in ETH resistance remain. TetR regulators can be dissociated from DNA through direct binding of small ligands (50). For MarR regulators, many of these ligands are aromatic compounds (50, 62). It remains unknown what biological conditions lead to transcriptional derepression of Mab_2648c targets, leading to upregulation of *mmpSL5* and possibly other genes. We speculate that these conditions are associated with ETH resistance, given that ETH exposure alone does not induce expression of either *MAB_2648c* or *mmpSL5* (Fig. 4B to E, Fig. S3A-B), suggesting that Mab_2648c may be associated with functions unrelated to drug resistance (50, 62, 63). The closest protein homologues of Mab_2649 and Mab_2650 in Mtb H37Rv are MmpS5 (Rv0677c, 38.57%) and MmpL5 (Rv0676c, 48.56%), respectively (64). MmpSL transporters are known to transport many lipids, some of which include TMM (MmpL3), PDIM (MmpL7), and sulfolipids (MmpL8) (65). MmpS/L4 and MmpL5 are associated with mycobactin and carboxymycobactin export, which are two of the major mycobacterial siderophores that allow for iron acquisition (65, 66). *Mtb* mutants defective in these transporters are unable to grow in low-iron environments (66). Although there are two MarR regulators in *Mtb* (Rv0042c and Rv0880), these do not share any homology with the MarR regulator identified in this work, suggesting that the mechanism by which Mab_2648c contributes to ETH resistance may be distinct from what is currently known in *Mtb* (61, 64).

It remains unknown as to whether spontaneous ETH resistance in *Mabs* is solely mediated by mutations in Mab_2648c. We examined three independent resistant clones, each obtained from a distinct ETH concentration. We were unable to examine a large range of concentrations for selection of resistant mutants as the agar MIC of ETH (65 μg/mL) was very close to the maximum solubility of ETH in agar (200 μg/mL). It is possible that spontaneous resistance to ETH may be associated with other genetic determinants, such as mutations in the promoter region of *inhA* (*MAB_2722c*) or mutations in potential bio-activators of ETH (*MAB_0103*, *MAB_0985*, *MAB_3967*) (23). The identification of 96 genes with insertions that conferred a growth advantage in the presence of ETH (Table S2) suggests that it is possible to obtain spontaneous mutations in genes independent of *MAB_2648c*.

Identifying few overlapping hits in our Tn-Seq screens with CLR and ETH was surprising. While ETH is known to target cell envelope metabolism in *Mtb*, this remains to be determined in *Mabs*. CLR synergizes with the cell wall targeting antibiotic vancomycin against *Mabs in vitro* (67), although the mechanisms responsible for this synergy remain unknown. We found that a combination of CLR and ETH was indifferent against *Mabs* (Table S4). Interestingly, Tn insertions in genes that conferred a growth disadvantage in the presence of CLR and ETH independently (*MAB_1055* and *MAB_0177*) encode proteins that are predicted to localize to the cell envelope (Fig. 5F). It is possible that a combinatorial action of CLR and ETH can lead to cell surface changes that makes the mycobacterial envelope more permeable to antibiotic entry. Although we did not observe synergy between CLR and ETH against WT *Mabs*, examining this combinatorial action in the absence of *MAB_1055* and *MAB_0177* may display a synergistic effect. Our collective findings in this work can be used to broaden our knowledge on identifying genetic determinants of ETH and other clinically relevant antibiotics. Identifying a gene that seems to uniquely confer resistance to ETH, but not other unrelated antibiotics, suggest that intrinsic drug resistance in *Mabs* may result from a multitude of genetic mechanisms.

## MATERIALS AND METHODS

### Construction of bacterial strains and growth conditions.

*Mabs* strain ATCC 19977 was grown in Middlebrook 7H9 (BD) media supplemented with albumin-dextrose-saline (ADS). For growth on solid media, *Mabs* was cultured on LB agar (BD) or Middlebrook 7H10 (BD) where indicated. Clinical strains were obtained from Chao Qi (Northwestern University). The following antibiotics were supplemented when appropriate for *Mabs*: hygromycin B (Invitrogen, 125 μg/mL for liquid media; 1 mg/mL for solid media), zeocin (InvivoGen, 50 μg/mL), and kanamycin (Sigma, 150 μg/mL for liquid media; 100 μg/mL for solid media). To favor the generation of S morphotype *Mab*s, both liquid and solid media were supplemented with Tween 80 (Sigma, 0.05% vol/vol). E. coli was grown in LB supplemented with the following antibiotics when appropriate: hygromycin B (125 μg/mL), zeocin (50 μg/mL), and kanamycin (50 μg/mL). Deletion of *MAB_2648c* and *MAB_2649-2650* was achieved using Oligonucleotide-Mediated Recombineering followed by Bxb1 Integrase Targeting (ORBIT) as previously described (68). We adapted the original ORBIT protocol for utilization in *Mabs*. Briefly, for each target gene, an oligonucleotide containing an *attP* site flanked by 60 to 80 bp of sequence homology surrounding each target gene was constructed and coelectroporated (2.5 kV, 25 μF, and 1,000 Ω) with the payload plasmid pKM496 into bacteria expressing genes from the plasmid pKM444 (68). All electroporations were conducted with 385 μL of bacteria washed three times with 10% glycerol at an optical density of 600 nm (OD600nm) of 0.1 to 0.3. Approximately 67 ng of target oligonucleotides (Integrated DNA Technologies) were used with and without 100 ng of pKM496 for electroporations using cuvettes containing 0.2 cm gaps (Bio-Rad). Following electroporation, bacteria were rinsed with 2 mL of 7H9 and allowed to recover at 37°C with shaking for 16 h before plating on LB supplemented with zeocin (50 μg/mL) to select for deletion mutants. PCR amplification was used to check for the correct recombination event, which was confirmed with Sanger sequencing.

### Tn-Seq screening, gene essentiality calls, and statistical analyses.

The transposon (Tn) library for *Mabs* was constructed as previously described for other mycobacterial species (40). For screening, an aliquot of the *Mabs* Tn library was grown in 150 mL of 7H9 broth until log phase (OD600nm 0.5 to 1.0). The Tn library was diluted to an OD_600_ of 0.01 (~5 × 10^6^ CFU/mL) in 10 mL of 7H9 with and without ETH (TCI Chemicals, 16 μg/mL), CLR (Ambeed, 1 μg/mL), and AMK (Ambeed, 8 μg/mL). All cultures were prepared in triplicate. Bacteria were grown at 37°C for 24 h, harvested by centrifugation, and washed twice with fresh 7H9 broth. Two mL of bacteria containing 1.5 × 10^4^ CFU/mL were plated on LB agar supplemented with kanamycin (100 μg/mL) and Tween 80 (0.05%) on 245 mm square bioassay dishes (Corning). Plates were incubated at 37°C for 5 days and then colonies were scraped into 35 mL of 7H9 broth. Genomic DNA was extracted from the collected mixture as previously described. Genomic DNA was submitted to the UC Davis DNA Technologies Core and sequences enriched for the *Himar1* transposon were amplified on a HiSeq illumina platform (43). Sequence reads were mapped to the *Mabs* ATCC 19977 genome. Transposon-enriched sequence reads were then analyzed using TRANSIT software (44). Calls of essentiality were made by TRANSIT using the Gumbel method with the following settings: 10,000 samples, trim setting = 1, minimum read set to 1, replicates averaged. For conditional essentiality, the calls were made by TRANSIT using the resampling method with the following settings: 10,000 resampling, TTR normalization, included zeros, trimming the first and last 5% of genes, winsorized resampling, and site restricted resampling.

### Determination of MICs and synergy testing.

*Mabs* strains were grown to an OD600nm of 0.2 to 1.0 in 7H9 and diluted to an OD600nm of 0.01 with and without a 2-fold dilution series of antibiotics to be tested in 96 well, TC-treated plates in a final volume of 100 μL. Plates were incubated at 37°C for 4 days without shaking in tightly sealed, moist Tupperware containers to prevent evaporation. After 4 days, bacteria were fixed with an equal volume of 5% Formalin (Sigma) for safety reasons. Optical densities were then recorded at 600 nm using a SpectraMax M3 Microplate Reader (Molecular Devices). The MIC here is defined as the lowest concentration of antibiotic that inhibits 99% of bacterial growth. For synergy experiments, ETH MIC was determined as described above with and without the highest concentration of each antibiotic tested that has no effect on bacterial growth. For CFU (CFU) enumeration, bacteria were washed twice in antibiotic-free growth media and then 10-fold serial dilutions were prepared followed by plating on LB agar. CFU/mL was enumerated after 5 days of incubation at 37°C.

### Selection of spontaneous ETH resistant mutants followed by whole-genome sequencing.

To select for spontaneous resistant mutants, WT *Mabs* (1 × 10^8^ CFU) was plated on LB agar supplemented with three different inhibitory concentrations of ETH (128, 150, and 200 μg/mL), which represents 2-, 2.3-, and 3.1-times MIC. Plates were incubated at 37°C for at least 7 days before inspection of the plates. Spontaneous resistant clones were picked and grown in 7H9 broth. ETH resistance was validated in the selected clones using the MIC protocol described above. Genomic DNA was extracted from the clones and submitted to the UC Davis DNA Technologies Core for whole-genome sequencing on a NovaSeq platform. Sequence reads were mapped to the *Mabs* ATCC 19977 genome.

### Quantitative PCR (qPCR).

Bacteria at an OD600nm of 0.1 were treated with and without ETH (16 μg/mL). At 3 and 11 h posttreatment, bacteria were harvested at 3,500 rpm for 10 min and RNA was extracted using TRIzol (Invitrogen) and purified using the RNeasy minikit (Qiagen) following the manufacturer’s protocol. Total RNA was reversed transcribed to cDNA using the Superscript III first-strand synthesis system (Invitrogen) following the manufacturer’s protocol. For each primer pair, 10-fold serial dilutions of cDNA were prepared for generation of standard amplification curves on a CFX Connect-Real Time PCR Detection System (Bio-Rad). Fluorescence was detected using the SsoAdvanced Universal SYBR green Supermix (Bio-Rad). cDNA synthesis reactions without RT were included in parallel to control for genomic DNA contamination. Expression levels were normalized to *sigA* (MAB_3009) before calculating relative expression levels using the delta-delta C_T_ method (2^-ΔΔC^_T_).

### Plasmids.

One thousand bp upstream of *MAB_2648c*, the *MAB_2648c* coding sequence, and 200 bp downstream of *MAB_2648c* were PCR amplified using Q5 DNA polymerase (New England Biolabs) and cloned into the DraI and HindIII sites of the integrative vector pMV306 (69), generating pRR107. pRR107 was electroporated into Δ*MAB_2648c* as described above and selected using kanamycin. The *mmpS5-L5* coding sequence along with 200 bp downstream of *mmpL5* were PCR amplified using Q5 DNA polymerase and cloned downstream of the anhydrotetracycline (ATc) inducible promoter of pUV15LD (55), generating pRR115. pRR115 was electroporated into both WT and Δ*MAB_2648c* bacteria and selected using Hygromycin B. To check for mycobacterial clones carrying desired plasmids, individual colonies were picked into 10 μL of sterile, nuclease-free water and heat-killed at 80°C for 1 h. 2 μL of heat-killed bacteria was used as a template for PCR using plasmid-specific primers.
