# Supplementary material for: MarR-Dependent Transcriptional Regulation of mmpSL5 Induces Ethionamide Resistance in Mycobacterium abscessus
Source: Antimicrob Agents Chemother. 2023 Mar 29;67(4):e01350-22. doi: 10.1128/aac.01350-22 (PMC10112066; doi:10.1128/aac.01350-22)
Supplement: Supplemental file 1 — Supplemental material. Download aac.01350-22-s0001.pdf, PDF file, 0.8 MB [file aac.01350-22-s0001.pdf]

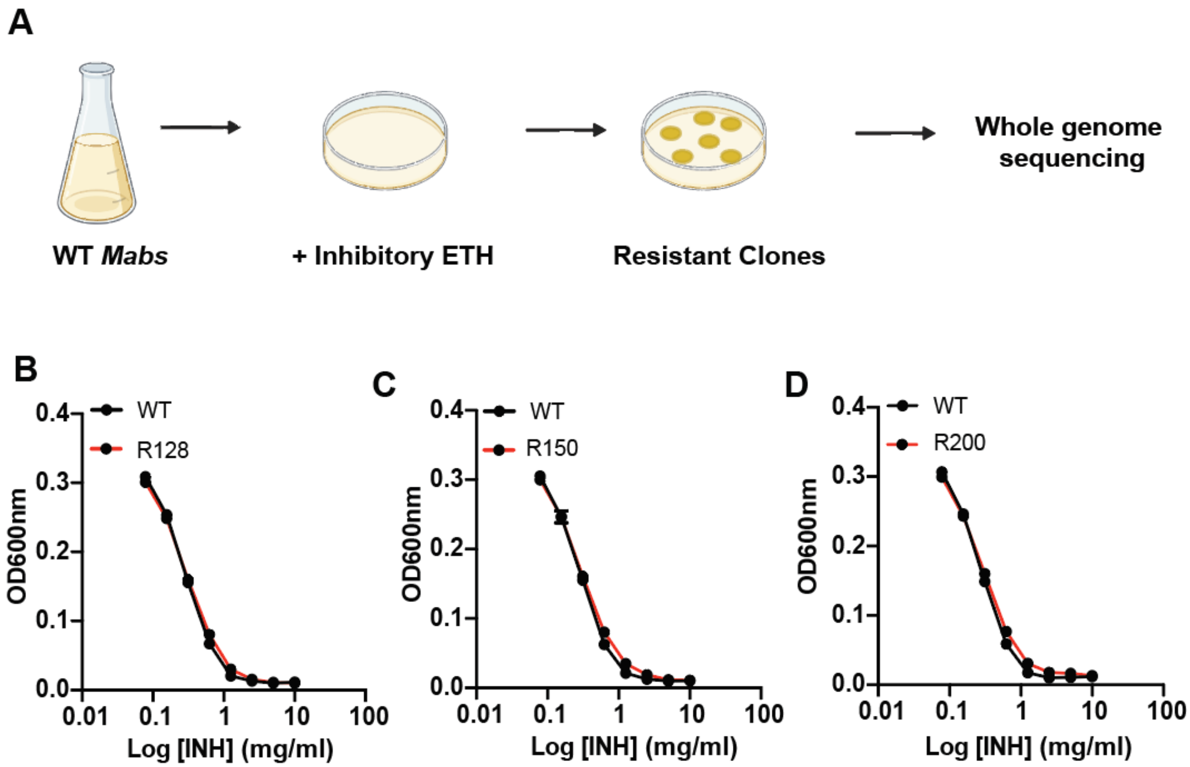

<u>Figure S1.</u> Spontaneous ETH resistance does not confer cross-resistance to INH. (A) WT *Mabs* was plated in the presence of inhibitory ETH to generate resistant bacteria, which were then analyzed by whole genome sequencing. (B-D) Isoniazid (INH) dose-response curves generated against isolated ETH-resistant clones (R128, R150, and R200). Experiments are representative of at least two biological replicates. Error bars represent standard deviation.

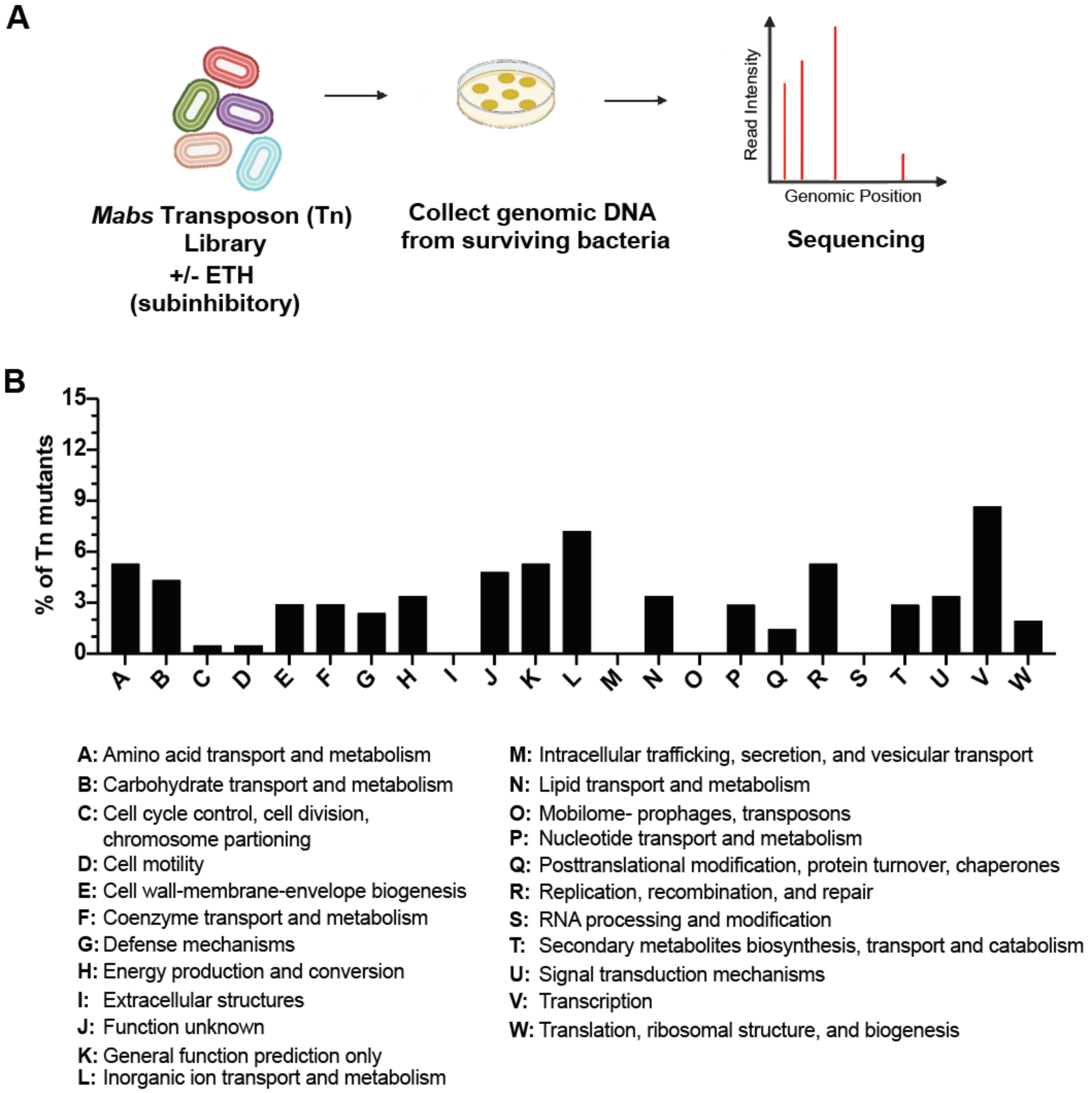

Figure S2. Cluster of Orthologous Group (COG) analysis of transposon mutants exposed to ETH. (A) A library of transposon mutants was treated with and without ETH. Surviving bacteria were then plated, genomic DNA collected, and transposon-enriched sequences analyzed by whole genome sequencing. (B) Percentage of transposon mutants represented in the indicated COG categories. Gene hits without any COG annotations are not shown.

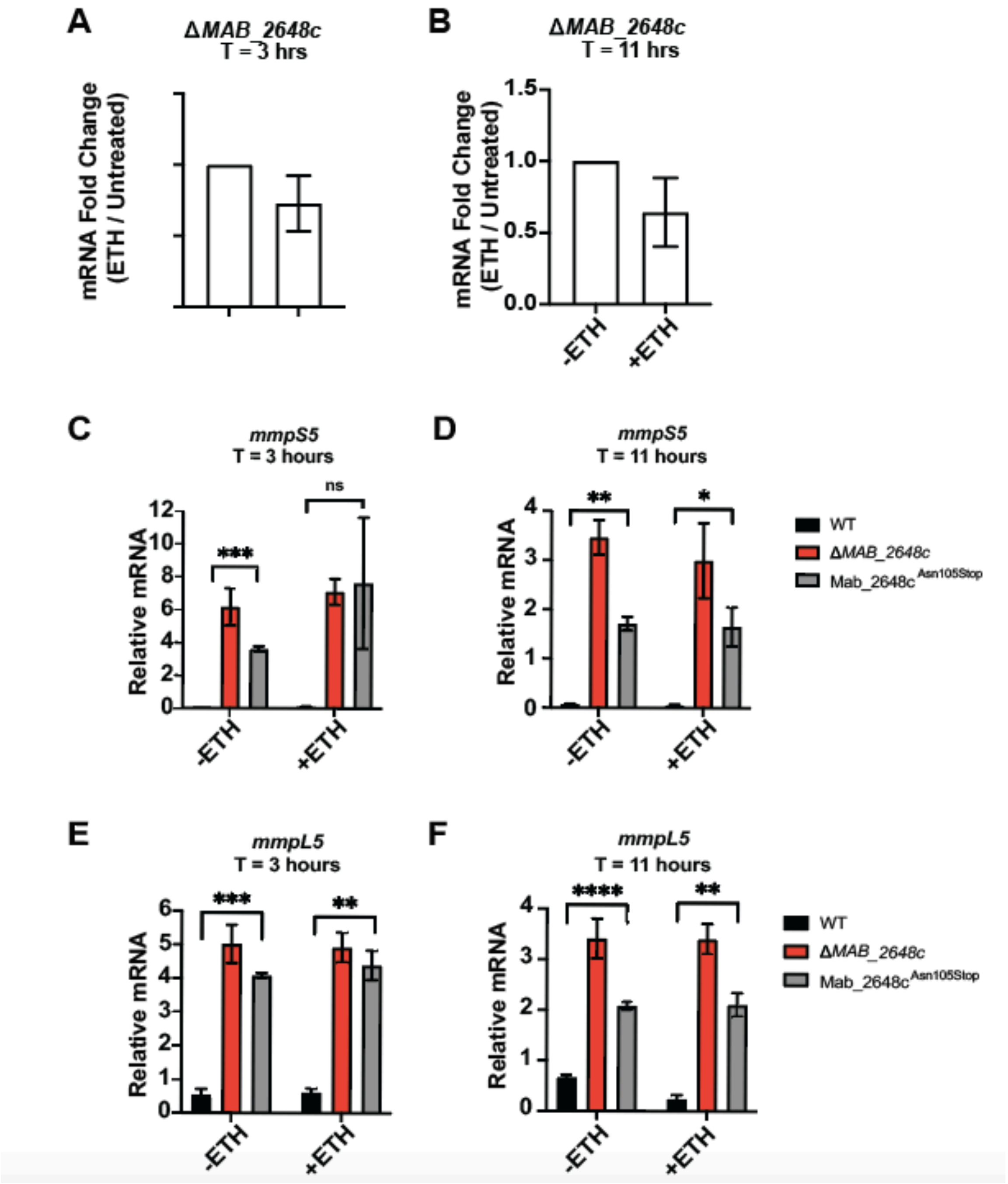

<u>Figure S3.</u> *MAB_2648c* is not induced in the presence of ETH. (A-B) Gene expression levels of *MAB_2648c* in WT *Mabs* in the presence and absence of ETH after 3 (A) and 11 hours (B) of exposure. (C-F) Gene expression levels of *mmpS5* and *mmpL5* in WT, Δ*MAB_2648c*, and MAB_2648c ^{Asn105Stop} bacteria in the presence and absence of ETH at the indicated time points. Experiments are representative of at least two biological replicates. Error bars represent standard deviation. no significance (ns); $p < 0.05$ (*); $p < 0.01$ (**); $p < 0.001$ (***); $p < 0.0001$ (****).

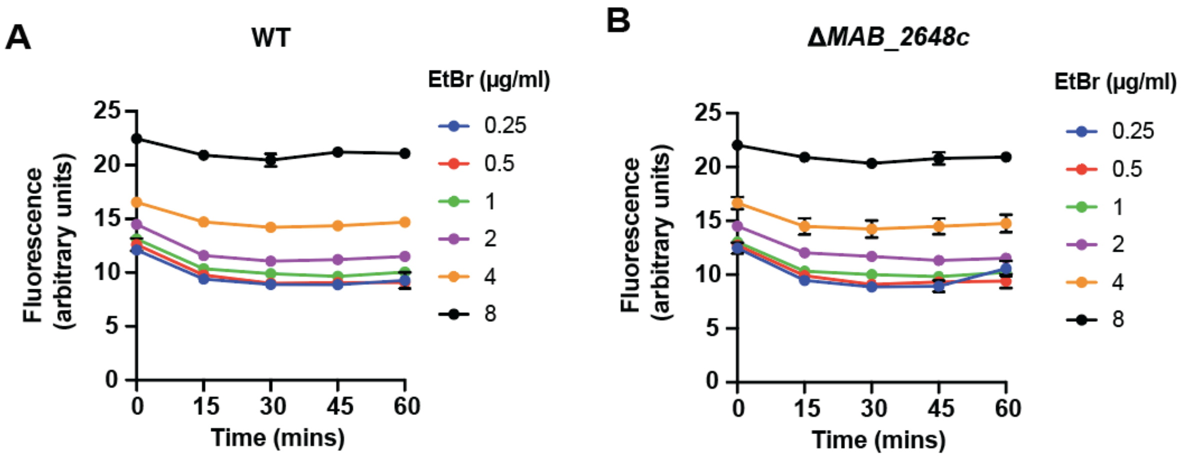

<u>Figure S4.</u> Loss of Mab_2648c activity does not lead to changes in Ethidium Bromide (EtBr) accumulation. WT (A) and Δ*MAB_2648c* (B) *Mabs* were treated with two-fold dilutions of EtBr (0.25 – 8 µg/ml) in PBS supplemented with 0.4% glucose (pH 7.4) and fluorescence measured for 60 minutes at 37°C (excitation wavelength: 530 nm; emission wavelength: 585 nm).

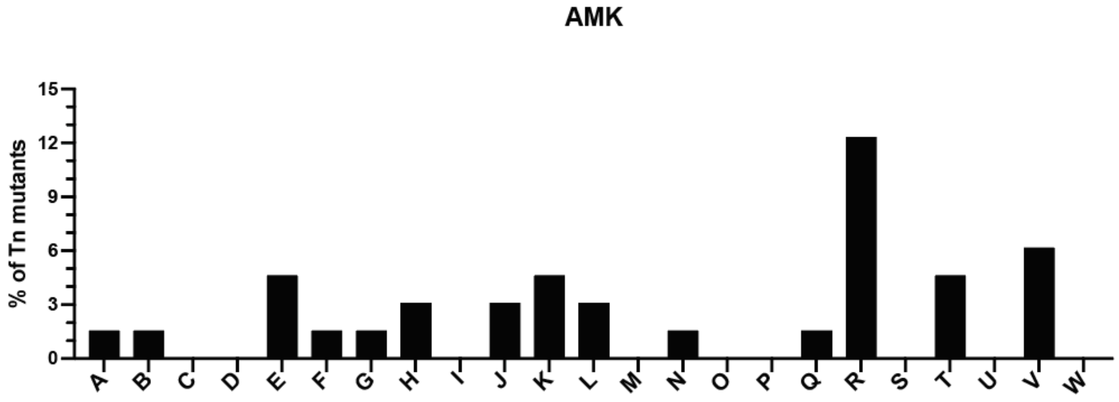

A: Amino acid transport and metabolism

B: Carbohydrate transport and metabolism

C: Cell cycle control, cell division, chromosome partioning

D: Cell motility

E: Cell wall-membrane-envelope biogenesis

F: Coenzyme transport and metabolism

G: Defense mechanisms

H: Energy production and conversion

I: Extracellular structures

J: Function unknown

K: General function prediction only

L: Inorganic ion transport and metabolism

M: Intracellular trafficking, secretion, and vesicular transport

N: Lipid transport and metabolism

O: Mobilome- prophages, transposons

P: Nucleotide transport and metabolism

Q: Posttranslational modification, protein turnover, chaperones

R: Replication, recombination, and repair

S: RNA processing and modification

T: Secondary metabolites biosynthesis, transport and catabolism

U: Signal transduction mechanisms

V: Transcription

W: Translation, ribosomal structure, and biogenesis

Figure S5. COG analysis of transposon mutants exposed to AMK. Percentage of transposon mutants exposed to AMK represented in the indicated COG categories. Gene hits without any COG annotations are not shown.

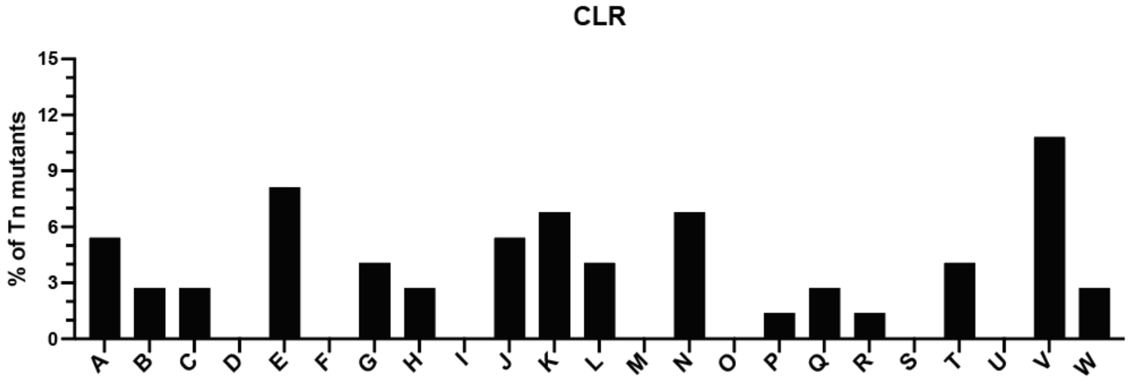

A: Amino acid transport and metabolism

B: Carbohydrate transport and metabolism

C: Cell cycle control, cell division, chromosome partioning

D: Cell motility

E: Cell wall-membrane-envelope biogenesis

F: Coenzyme transport and metabolism

G: Defense mechanisms

H: Energy production and conversion

I: Extracellular structures

J: Function unknown

K: General function prediction only

L: Inorganic ion transport and metabolism

M: Intracellular trafficking, secretion, and vesicular transport

N: Lipid transport and metabolism

O: Mobilome- prophages, transposons

P: Nucleotide transport and metabolism

Q: Posttranslational modification, protein turnover, chaperones

R: Replication, recombination, and repair

S: RNA processing and modification

T: Secondary metabolites biosynthesis, transport and catabolism

U: Signal transduction mechanisms

V: Transcription

W: Translation, ribosomal structure, and biogenesis

Figure S6. COG analysis of transposon mutants exposed to CLR. Percentage of transposon mutants exposed to CLR represented in the indicated COG categories. Gene hits without any COG annotations are not shown.

Gene Name	Tb Locus	Essentiality (TB)	Mabs Locus	Essentiality (Mabs)
MmpL3	Rv0206c	ES	MAB_4508	ES
FabH	Rv0533	NE	MAB_1141c	NE
HadA	Rv0635	ES	MAB_3898c	NE
HadB	Rv0636	ES	MAB_3897c	NE
HadC	Rv0637	NE	MAB_3896c	NE
MabA	Rv1483	ES	MAB_2723c	NE
InhA	Rv1484	ES	MAB_2722c	ES
FabD	Rv2243	ES	MAB_1879c	NE
AcpM	Rv2244	ES	MAB_1878c	ES
KasA	Rv2245	ES	MAB_1877c	ES
KasB	Rv2246	NE	MAB_2028	NE
AccD6	Rv2247	GD	MAB_1876c	NE
CmrA	Rv2509	ES	MAB_1537c	NE
FAS-I	Rv2524c	ES	MAB_1512	ES
AccA3	Rv3285	ES	MAB_3643	ES
AccD4	Rv3799c	ES	MAB_0181	ES
Pks13	Rv3800c	ES	MAB_0180	ES
FadD32	Rv3801c	ES	MAB_0179	ES

Fas-II
FAS-I/central carbon metabolism
Mycolate condensation and maturation
Funneling of intermediates to FAS-II

Table S2. Most mycolic acid biosynthetic genes are essential in both *Mtb* and *Mabs.* ES, essential; NE; not essential; GD, growth defect

Antibiotic	WT MIC (µg/ml)	Δ*MAB_2648c* MIC (µg/ml)
Amikacin	2	2
Bedaquiline	0.25	0.25
Cefoxitin	4	4
Clofazimine	>16	>16
Ethambutol	32	32
Isoniazid	1250	1250
Thiacetazone	> 500	>500
Imipenem	4	4
p-aminosalicylic acid	> 160	> 160
Moxifloxacin	1	1
Doxycycline	> 64	> 64
Clarithromycin	4	4

Table S3. Comparison of MIC values in WT and Δ*MAB_2648c Mabs* to functionally diverse antibiotics.

Antibiotic	ETH MIC (µg/ml)
AMK	8
CLR	8
MFX	8
CEF	4
IPM	4

Table S4. ETH MIC values in WT *Mabs* in the presence of each indicated antibiotic. AMK: Amikacin; CLR: Clarithromycin; MFX: Moxifloxacin; CEF: Cefoxitin; IPM: Imipenem
